# Revisiting the origin and diversification of vascular plants through a comprehensive Bayesian analysis of the fossil record

**DOI:** 10.1111/nph.13247

**Published:** 2015-01-26

**Authors:** Daniele Silvestro, Borja Cascales‐Miñana, Christine D. Bacon, Alexandre Antonelli

**Affiliations:** ^1^ Department of Biological and Environmental SciencesUniversity of GothenburgCarl Skottsbergs gata 22BSE‐413 19GöteborgSweden; ^2^ CNRSUMR Botanique et Bioinformatique de l'Architecture des Plantes (AMAP)MontpellierF‐34000France; ^3^ Laboratório de Biología Molecular (CINBIN)Department of BiologyUniversidad Industrial de SantanderBucaramangaColombia; ^4^ Gothenburg Botanical GardenCarl Skottsbergs gata 22ASE‐413 19GöteborgSweden; ^5^ Present address: PPPDépartement de GéologieUniversité de LiègeAllée du 6 AoûtB18 Sart TilmanB4000LiègeBelgium

**Keywords:** biodiversity changes, diversification, floristic turnover, mass extinction, plant fossils, PyRate

## Abstract

- Plants have a long evolutionary history, during which mass extinction events dramatically affected Earth's ecosystems and its biodiversity. The fossil record can shed light on the diversification dynamics of plant life and reveal how changes in the origination–extinction balance have contributed to shaping the current flora.
- We use a novel Bayesian approach to estimate origination and extinction rates in plants throughout their history. We focus on the effect of the ‘Big Five’ mass extinctions and on estimating the timing of origin of vascular plants, seed plants and angiosperms.
- Our analyses show that plant diversification is characterized by several shifts in origination and extinction rates, often matching the most important geological boundaries. The estimated origin of major plant clades predates the oldest macrofossils when considering the uncertainties associated with the fossil record and the preservation process.
- Our findings show that the commonly recognized mass extinctions have affected each plant group differently and that phases of high extinction often coincided with major floral turnovers. For instance, after the Cretaceous–Paleogene boundary we infer negligible shifts in diversification of nonflowering seed plants, but find significantly decreased extinction in spore‐bearing plants and increased origination rates in angiosperms, contributing to their current ecological and evolutionary dominance.

## Abstract 2

See also the Commentary by Niklas

## Introduction

Understanding the tempo and mode of species diversification – that is, the interplay between speciation and extinction – has engaged naturalists for centuries (e.g. Darwin, 1859). In particular, the evolution of vascular plants (tracheophytes) still remains an area of active research (e.g. Boulter *et al*., 1988; Raubeson & Jansen, 1992; Kenrick & Crane, 1997; Pryer *et al*., 2001). In the pioneering work by Niklas *et al*. (1983) four time periods were identified as phases of increased land plant diversification: the Devonian, Carboniferous–Permian, Triassic–Jurassic and Early Cretaceous. Land plants also went through four of the five major extinction events (the ‘Big Five’; Jablonski, 2005) identified from marine organisms at the Frasnian–Famennian, the Permian–Triassic, the Triassic–Jurassic and the Cretaceous–Paleogene boundaries (Raup & Sepkoski, 1982).

The four phases of plant radiation described by Niklas *et al*. (1983) began with the Silurian–Devonian diversification and expansion of early land plants (embryophytes) (Edwards & Wellman, 2001; Edwards & Richardson, 2004; Wellman, 2014) and the evolution of early forests (Meyer‐Berthaud *et al*., 2010). The second phase was characterized by the dominance of lycophytes and Carboniferous tropical wetlands (Cleal, 2008a,b,c; Cleal *et al*., 2012) together with the diversification of early ‘gymnosperms’ (a nonmonophyletic group including several lineages, e.g. Cordaitales) and seed ferns (Anderson *et al*., 2007). The third phase encompasses the recovery from the Late Permian mass extinction event (Niklas, 1997; Rees, 2002) and the establishment of ‘gymnosperms’ as the dominant plant group (Anderson *et al*., 2007; Bonis & Kürschner, 2012). The last phase (Early Cretaceous) witnessed the radiation of angiosperms, which are today the world's most dominant plants in species richness and ecological differentiation (Crepet & Niklas, 2009; Friis *et al*., 2011). The broad patterns of vascular plant history can also be explained in terms of turnover of five major floras, also known as the Rhyniophytic (= Eotrachyophytic), Eophytic, Paleophytic, Mesophytic and Cenophytic Floras (Cleal & Cascales‐Miñana, 2014). Following this scheme, the first two represent the early evolutionary trends of plant life, whereas the Paleophytic, Mesophytic and Cenophytic Floras represent the heyday of spore‐bearing plants (i.e. lycophytes and ferns), ‘gymnosperms’ and angiosperms, respectively (Cleal & Cascales‐Miñana, 2014).

Except for the origin of angiosperms, the majority of biological events that have characterized plant diversification are rooted in the Paleozoic. Some of the most crucial events include the invasion of land by early plants (the ‘terrestrialization process’; Kenrick & Crane, 1997; Vecoli *et al*., 2010), the heyday of Carboniferous tropical wetlands (Cleal *et al*., 2011), the reduction of arborescent lycophyte forests in the Pennsylvanian (Dimitrova *et al*., 2010; Cleal *et al*., 2012), and the major extinction of plant diversity at the Permian–Triassic boundary (Rees, 2002; McElwain & Punyasena, 2010; Cascales‐Miñana & Cleal, 2014). Considerable advances have been made recently in our understanding of these key events, and their impact on past ecosystem dynamics. For instance, the fossil record from South China suggests that plant diversity dynamics during the Permian–Triassic were characterized by a gradual floral turnover, rather than a mass extinction like those in the coeval marine faunas (Xiong & Wang, 2011). Furthermore, fossil evidence suggests that diversity dynamics of land plants were considerably different from those of marine faunas and land vertebrates during the Devonian, but primary diversity changes affected all terrestrial organisms synchronously at the Devonian–Carboniferous boundary (Xiong *et al*., 2013 and references therein). Despite these recent advances, few studies have attempted a quantitative assessment of the origin and diversification dynamics of vascular plants following uniform criteria regarding taxonomic, temporal and spatial scales. This lack of formal, data‐driven approaches calls for a re‐evaluation of the unequivocal events of increased plant origination and extinction, which are supported by statistical significance. Ideally, such analysis should be done at the finest reliable taxonomic scale, under a global perspective, and using recent advances in paleobotany, as well as the most sophisticated and statistically robust analytical methods.

### Estimating the origin and diversification of plant groups using the fossil record

The plant fossil record has been primarily explored using range‐based counts (Cleal, 1993; Anderson *et al*., 2007), rather than occurrence‐based data. Age ranges include the first and last appearance of a taxon, whereas taxonomic occurrence data include finer detail of fossil individuals, such as abundance and geographic distribution. Range‐based approaches are commonly applied with standard datasets above the generic level to infer biodiversity and diversification trends across broad time intervals (e.g. for Phanerozoic trends; Sepkoski *et al*., 1981). By contrast, targeting finer‐scale diversification events (e.g. the Late Permian biotic crisis and its corresponding recovery period) requires the analytical implementation of sampling parameters from fossil occurrence data (e.g. Alroy *et al*., 2001). Both approaches to exploring the fossil record are valid, but the use of occurrence data opens the door to a battery of methods to analyze data and test hypotheses in a more robust statistical framework.

A new method, PyRate, was developed to infer the rates of speciation (or origination for higher taxonomic ranks) and extinction and their variation through time, within a Bayesian probabilistic framework (Silvestro *et al*., 2014b). The method is capable of handling fossil occurrence data (i.e. not only first and last appearances) and dealing with the inherent incompleteness of the fossil record. PyRate simultaneously estimates rates of origination, extinction and preservation, as well as the lifespan of each taxa in the dataset. The method thus takes into account the uncertainties associated with the fossil preservation process.

A major goal in plant evolutionary research is to determine the temporal origin of major groups. Usually the oldest known fossil evidence of a given lineage is assumed to closely correspond to its true time of origin. However, this can be controversial due to the lack of consensus on the most reliable type of fossil (e.g. pollen data vs fossil leaves; Kenrick *et al*., 2012), or because of differences in the results obtained from fossil data and from dated molecular phylogenies (Doyle & Endress, 2010). In fact, dated phylogenies of extant taxa rely heavily on the selection of fossil calibrations (Sauquet *et al*., 2012) and the associated prior distributions (Ho & Phillips, 2009; Heath, 2012). PyRate is able to produce statistically robust estimates of origination times, because it uses the complete fossil record (i.e. not only the oldest fossil) and incorporates a preservation process. Therefore, it can be used to reduce the potential biases deriving from arbitrarily defined prior distributions.

Here, we characterize the main radiations and biotic crises of plants within the Bayesian framework implemented in PyRate. From this, we (1) infer the fluctuations of origination and extinction rates through time for vascular plants (as a whole as well as subdivided into three main vegetation groups), (2) evaluate the effect of the largest widely recognized mass extinction events on both origination and extinction rates, and (3) derive the age of origin of vascular plants, seed plants and angiosperms, providing posterior distribution curves for their ages rather than point estimates, which can be directly used as prior calibrations in molecular dating analyses.

## Materials and Methods

### Data compilation

This study is based on plant fossil occurrences downloaded from the Paleobiology Database (PBDB; http://paleobiodb.org/cgi-bin/bridge.pl) on 6 March 2014. We performed multiple search queries in order to obtain the most comprehensive dataset of the plant fossils, on a global level. The approach that captured the maximum number of records involved querying the database for all described plant classes and orders individually. With this approach we obtained 35 666 entries, which spanned most of the Phanerozoic (the oldest occurrences dating back to *c*. 424 million yr ago (Ma)). We included all occurrences that were identified to the species level as well as those identified only at the genus level but without specific assignation (e.g. ‘*Psilophyton* sp.’). We did not revise fossil identifications provided by the PBDB. Several culling procedures are normally used to avoid including incompletely identified taxa, such as searching and excluding entries identified with aff., cf., sp. or other modifications to binomial species names (Janevski & Baumiller, 2009). We initially explored the effect of excluding such records from the analyses, but because we found no differences in genus‐level diversity patterns we opted for using all available records. The dataset thus includes records from all lithologies, environments and continents that were freely available from the PBDB, including their estimated ages in millions of years (Myr). Raw data are available as supplementary materials (Supporting Information Tables S1, S2).

It is well known that the plant fossil record is highly fragmentary, not least for plants (Cleal & Thomas, 2010). This is further complicated by the incomplete nature of fossilized plant organs, varying quality of preservation, and our incomplete knowledge on morphological evolution. These factors lead to difficulties in confidently assessing taxonomic affinity, and the existence of many fossils of uncertain taxonomic placement or ‘*incertae sedis’*. To minimize such problems we analyzed occurrence data at the genus level assuming that this level best captures the long‐term and global‐level dynamics of plant diversification (Rees, 2002; Wang *et al*., 2010; Xiong & Wang, 2011; Xing *et al*., 2014). An additional problem in the plant fossil record is that a single genus may be represented in several ‘fossil‐genera’ described from different plant remains or organs (e.g. in arborescent lycophytes; Cleal *et al*., 2012). This might have the effect of overestimating plant diversity from some time intervals (e.g. the Carboniferous). Although our dataset includes several different types of plant remains (e.g. leaves, fertile and sterile axes, seed, fruits and roots), leaf samples represent > 70% of all the occurrences. Therefore, we considered the discrepancies between genera and fossil‐genera as unlikely to alter the major fluctuations of plant diversity.

There are several practical reasons for avoiding species‐level analyses. First, excluding all fossils without assignation to the species level would reduce the full dataset by 47%. Second, > 50% of the species we compiled are known from a single occurrence, which makes it difficult to estimate accurate preservation rates regardless of the methodological approach used. Lastly, species‐level data should arguably be more sensitive to floristic variation due to regional endemism and fossil preservation biases. The interpretation and analytical incorporation of regional variations of species diversity is often problematic, as it could either derive from true biodiversity differences or reflect the fact that regional morphological differences are often described as new species in the absence of more thorough evidence. Thus, we consider that genus‐level analyses constitute a robust way of exploring the diversification dynamics of the plant fossil record.

Plant macrofossils (e.g. whole flowers, fruits, seeds, leaves) are commonly assumed to be the most reliable source of data to study plant evolution, because they usually contain many taxonomically useful characters and can therefore be more easily and reliably assigned to different plant groups (Wang *et al*., 2010). By contrast, microfossils (pollen and spores) often lack a sufficient amount of diagnostic synapomorphies that facilitate taxonomic identification at the generic level. We therefore subsampled our original dataset to include only macrofossils (i.e. > 60% of the original dataset) in the analyses.

Analyses of diversification should ideally be performed on monophyletic groups, that is, clades in a phylogenetic sense. However, although monophyly assessment is straightforward in phylogenetic analyses based on molecular data, many questions remain on the phylogenetic placement of several fossil taxa. In addition, restricting the analyses to clades would require splitting the already fragmentary fossil data into smaller subsets, reducing analytical power and precision. We therefore categorized vascular plant fossil‐genera into three major groups, distinguished by their functional ecology, morphology and relationships (following Kenrick & Crane, 1997; Tomescu, 2009; Friis *et al*., 2011; Hao & Xue, 2013): (1) spore‐bearing plants (i.e. early tracheophytes, rhyniophytes, lycophytes, sphenophytes, early euphyllophytes, progymnosperms, ferns and related plants, but excluding bryophytes); (2) nonflowering seed plants (i.e. ‘gymnosperms’ including Cycadidae, Ginkgoidae, Gnetidae, Pinidae, Bennettitales, *Caytonia* and seed ferns); and (3) flowering seed plants (i.e. angiosperms). Fossil occurrences were assigned to the different categories based on the supra‐generic taxonomic information provided by the PBDB and, when available, using the *Index Nominum Genericorum* (Smithsonian Institution, http://botany.si.edu/ing/). We matched the names of fossil‐genera in our dataset with those listed at *The Plant List* (http://www.theplantlist.org/) to identify which taxa are extant.

The final dataset of macrofossils included 22 415 occurrences assigned to 443 genera, of which 263 are extinct and 180 are extant. The fossil occurrences were evenly distributed across the three plant groups described above: spore‐bearing plants (34%), nonflowering seed plants (34%) and angiosperms (32%). Angiosperms encompassed 58% of the diversity (with 257 genera) whereas the remaining genera were evenly distributed among the other two plant groups. The large majority of fossil occurrences included temporal ranges (minimum and maximum ages), which typically derive from the upper and lower temporal boundaries of the stratigraphic units to which fossils were assigned. In our dataset, temporal ranges spanned on average 12.7 Myr with a standard deviation of 12.6 Myr. In order to account for these dating uncertainties in the analyses of origination and extinction rates we considered the temporal ranges as uniform distributions and, from these, we randomly resampled the ages of each fossil occurrence following Silvestro *et al*. (2014b). We generated 100 datasets using this randomization approach and ran the analyses on all replicates.

### Diversification rate analyses

We carried out the analyses of plant diversification using a hierarchical Bayesian model to infer the temporal dynamics of origination and extinction (Silvestro *et al*., 2014b. This approach, implemented in the program PyRate (http://sourceforge.net/projects/pyrate/; Silvestro *et al*., 2014a), uses as input data all fossil occurrences that can be assigned to a taxon, in this case fossil‐genera, to jointly model the preservation and diversification processes. The preservation process infers the individual origination and extinction times of each taxon based on all fossil occurrences and on an estimated preservation rate (expressed as expected occurrences per taxon per Myr). This process is important because the first and last appearances of a taxon are likely to underestimate the true extent of its lifespan (Liow & Stenseth, 2007). The origination and extinction rates are inferred from the estimated lifespans of taxa based on a birth–death process, which models the diversification dynamics underlying diversity changes and constitutes the prior over the origination and extinction times (Silvestro *et al*., 2014b). In summary, the main parameters jointly estimated by this approach are: preservation rate and its degree of heterogeneity; origination and extinction times; and origination and extinction rates, which can vary through time. All of these parameters are sampled from their posterior distributions using a Markov Chain Monte Carlo (MCMC) algorithm, yielding parameter estimates and credible intervals, here calculated as 95% highest posterior density (HPD) intervals.

Compared to the original model described by Silvestro *et al*. (2014a,b), we introduced some modifications that better comply with the type of data and the aims of this study, which is mainly focused on variation in origination and extinction at global scale and large temporal ranges. We used here an homogeneous Poisson process (HPP) of preservation instead of the original ‘hat’‐shaped nonhomogeneous process (NHPP; Liow *et al*., 2010; Silvestro *et al*., 2014b). Although we believe the NHPP robustly describes fossil abundance in well‐preserved marine or mammalian species, we consider the HPP as a more appropriate assumption in the case of genus‐level plant fossil data due to potentially sparse sampling. We also accounted for varying preservation rates across taxa using the Gamma model, that is, with gamma‐distributed rate heterogeneity (Silvestro *et al*., 2014b). Because of the large number of occurrences analyzed and of the vast timescale considered, we used here eight rate categories to discretize the gamma distribution, instead of the default four, to accommodate the potential for more variability of preservation rates across taxa.

Furthermore, we broke down the birth–death process into segments of time, defined by the epochs of the stratigraphic geological timescale (Cohen *et al*., 2013) and estimated origination and extinction rates within these intervals. We adopted this solution as an alternative to the algorithms implemented in the original PyRate release that jointly estimate the number of rate shifts and the times at which origination and extinction undergo a shift. The estimation of origination and extinction rates within fixed time intervals improved the mixing of the MCMC and allowed us to obtain an overview of the general trends of rate variation throughout a long timescale, that is, almost the entire the Phanerozoic, within reasonable computational effort (a few days on a computer cluster). We emphasize that both the preservation and the birth–death processes are still modeled in continuous time and are not based on boundary crossings. Thus, the origination and extinction rates are measured as the expected number of origination and extinction events per lineage per Myr. One potential problem in fixing *a priori* the number of rate shifts is overparameterization. We overcame this issue by assuming that the rates of origination and extinction are part of two families of parameters following a common prior distribution, with parameters estimated from the data using hyper‐priors (Gelman, 2004). Thus, we used Cauchy prior distributions centered at 0 and with scale (hyper‐)parameters estimated from the data in the MCMC. The use of hyper‐priors is a convenient way to reduce the subjectivity in defining the priors and to reduce the effective parameter space, thus limiting the risks of overparameterization (Gelman, 2004).

**Figure 1:**
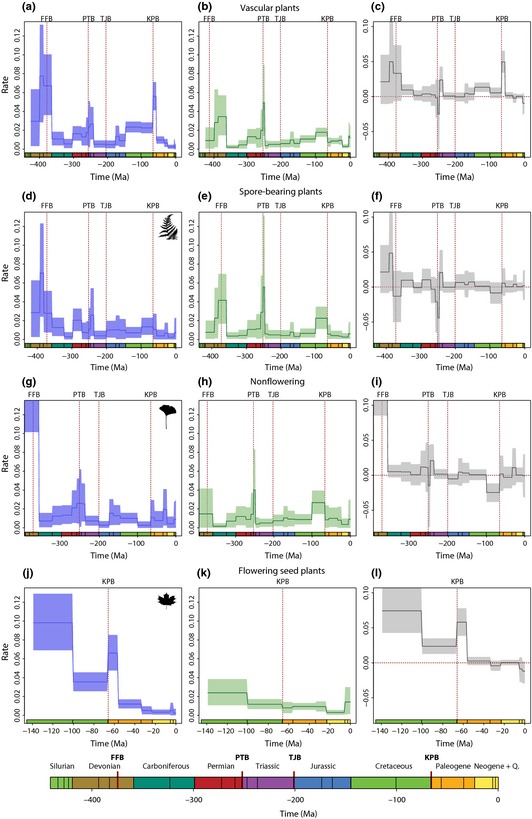
Generic‐level diversification analysis of all vascular plants. Origination (blue) and extinction (green) rates were estimated using the Bayesian approach implemented in PyRate (Silvestro *et al*., 2014a,b) within time bins defined as epochs of the geologic timescale (shown at the bottom). The timescale (*x‐*axis) is given in million years ago (Ma). Solid lines indicate mean posterior rates, whereas the shaded areas show 95% HPD intervals. The diversification dynamics were estimated for vascular plants as a whole (a–c), spore‐bearing plants (d–f), nonflowering seed plants (g–i) and flowering seed plants (angiosperms; j–l). Net diversification rates (gray) are defined as origination minus extinction. The dashed lines indicate the major mass extinction events widely recognized: at the Frasnian–Famennian (FFB), Permian–Triassic (PTB), Triassic–Jurassic (TJB) and Cretaceous–Paleogene (KPB) boundaries. Plant silhouettes were obtained from http://phylopic.org.

**Figure 2:**
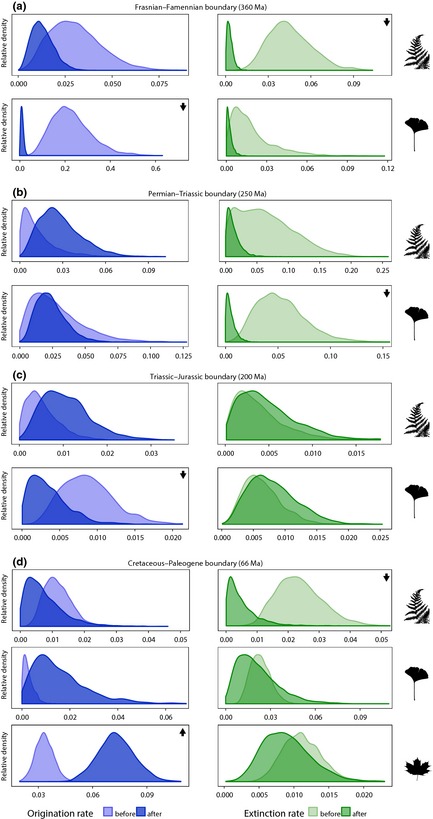
Origination and extinction rates of plants across major events of global biodiversity turnover, the Frasnian–Famennian (a), Permian–Triassic (b), Triassic–Jurassic (c) and Cretaceous–Paleogene (d) boundaries. The time of the events is provided in million years ago (Ma). These plots show the sampled posterior distributions of origination (blue) and extinction (green) rates that were estimated before and after each event. Posterior distributions were standardized to relative densities for graphical purposes. Arrows on the top‐right corner of panels indicate significant rate increases (pointing up) or decreases (pointing down) based on the 95% HPD of the two distributions.

We ran PyRate for 5000 000 MCMC generations on each of the 100 randomly replicated datasets. After excluding the first 20% of the samples as burnin phase, we combined the posterior estimates of the origination and extinction rates across all replicates and used them to generate rates‐through‐time plots. Rates of two adjacent intervals were considered significantly different when the mean of one lay outside of the 95% HPD of the other, and vice versa. We looked at the marginal posterior distributions of origination and extinction rates through the largest extinction events documented in geological history, the so‐called ‘Big Five’ mass extinction events in life's history (Jablonski, 2005; McElwain & Punyasena, 2010). In particular, we examined the diversification dynamics at four of the ‘Big Five’ events (the Frasnian–Famennian, Permian–Triassic, Triassic–Jurassic and Cretaceous–Paleogene boundaries; see the Introduction section; Figs 1, 2). The Ordovician–Silurian extinction event occurred before the appearance of vascular plants and was therefore not considered. We focused on the magnitude of rate changes, their statistical significance and the uncertainty around those estimates. We measured extinction as the rate of disappearance of fossil‐genera, which are described based on morphology and might not always represent phylogenetic lineages. Thus, it was not possible with our data to discern potential events of pseudo‐extinction (i.e. when two fossil‐genera describe a single lineage evolving to a modified form).

We estimated the origination times of vascular plants, seed plants and flowering plants from the posterior distributions of the origination times of the groups analyzed, following Silvestro *et al*. (2014b). Based on the posterior samples of the ages of origin derived from all replicates, we identified the best‐fitting distribution by testing several distributions (uniform, normal, log‐normal, gamma and Laplace) under maximum likelihood. We emphasize that these estimates are derived after explicitly modeling the fossil preservation process, and accounting for the uncertainties around the estimated ages of the fossil occurrences. After choosing the best‐fitting distribution based on the Akaike Information Criterion (AIC; Akaike, 1974), we computed the estimated parameter values, which may be directly used for other studies as calibrations to date molecular phylogenetic trees using relaxed molecular clock models (e.g. Lepage *et al*., 2007). The estimated parameters and offset values are given following the parameterization used in BEAST (Drummond *et al*., 2012), one of the most popular programs for molecular dating. We implemented these fitting functions in the latest version of PyRate.

## Results

### Diversification dynamics

Our results show that the diversification history of vascular plants as a whole was characterized by several changes in global origination and extinction rates through time (Fig. 1a–c). The origination rates showed three main peaks, at the Middle Devonian, during the Early Triassic and at the early Paleogene (Fig. 1a). We estimated the highest extinction rates at the Paleozoic–Mesozoic boundary and during the Late Devonian. Overall, plants suffered less extinction during the Mesozoic than the Paleozoic; however, we inferred a progressive increase in extinction rates towards the Late Cretaceous (Fig. 1b). In terms of net diversification rates (origination minus extinction), we inferred strongly negative diversification at the earliest Triassic followed by a peak of positive rates in the Middle Triassic (Fig. 1c). The highest diversification values were detected in the Late Devonian and early Cenozoic. Diversification rates declined to negative values towards the present day.

Results of spore‐bearing plant diversification history (Fig. 1d–f) revealed three main episodes of origination at the Middle Devonian, the Early Permian and the Early–Middle Triassic. Origination rates declined progressively through the Late Devonian and Carboniferous. During the Mesozoic and Cenozoic, spore‐bearing plant diversity witnessed quite stable levels of origination with slightly higher rates in the Late Cretaceous (Fig. 1d). Spore‐bearing plants experienced phases of high extinction during the Late Devonian, Late Permian and Late Cretaceous (Fig. 1f). We detected a progressive increase in extinction through the Carboniferous and Permian, and the highest value was inferred at the Paleozoic–Mesozoic boundary (Fig. 1e). Consequently, the main fluctuations in net diversification rates were estimated in the Late Devonian and during the Permian–Triassic and Cretaceous–Paleogene transitions (Fig. 1f).

The diversification dynamics of nonflowering seed plants (Fig. 1g–i) showed very high origination rates during the Devonian, followed by a drastic drop at the Mississippian (Early Cretaceous). We then observed high turnover with a progressive increase in both origination and extinction values culminating at the late Paleozoic (Fig. 1g,h). Another origination peak was at the middle Jurassic (Fig. 1g), whereas a second maximum of extinction was detected at the Late Cretaceous (Fig. 1h). This was followed by diversity recovery during the Cenozoic with diversification rates increasing to non‐negative values (Fig. 1i).

Angiosperm diversification (Fig. 1j–l) was characterized by very high initial origination rates, progressively decreasing towards the present (Fig. 1j). The clade diversified under a high origination and comparatively low extinction rate, contributing to significantly positive net diversification lasting from its origin to the Paleocene. We found a maximum value of origination in the Early Cretaceous (Fig. 1j) and a second origination peak during the early Paleogene. Overall, extinction rates appear quite low and stable through time (Fig. 1k). Angiosperm net diversification rates show negative values at the Paleogene and at more recent times (Fig. 1l).

### Shifts in origination and extinction rates

We found a significant decrease (> 20‐fold) in the extinction rate of spore‐bearing plants following the Frasnian–Famennian boundary, which did not take place in similar magnitude among seed plants (Fig. 2a). Although for both spore‐bearing plants and nonflowering seed plants extinction rates strongly decreased (> 10‐fold) after the Permian–Triassic boundary, such decrease was found to be significant only in nonflowering seed plants (Fig. 2b), potentially as a result of a high degree of uncertainty around the posterior rate estimates (as reflected by the relatively large shaded area under their curves). Across the Triassic–Jurassic boundary we did not detect any notable changes of extinction rates in spore‐bearing and seed plants, but the origination rates in the latter group experienced a significant 3.6‐fold decrease (Fig. 2c). This suggests that the Frasnian–Famennian boundary event had a stronger impact on the extinction of spore‐bearing plants than on the extinction of other plant groups, whereas the Permian–Triassic boundary event mostly affected the extinction of seed plants. Likewise, the Frasnian–Famennian and the Triassic–Jurassic boundaries are preceded by periods of high origination, mainly for seed plant diversity. Interestingly, the transition between the Mesozoic and the Cenozoic (the Cretaceous–Paleogene boundary) involved significant changes in extinction rates only in spore‐bearing plants, in which the extinction rates decreased 10‐fold (Fig. 2d). The Cretaceous–Paleogene boundary was followed by a significant (2.2‐fold) increase in origination rates in angiosperms (Fig. 2e).

### Origin of major plant clades

**Figure 3:**
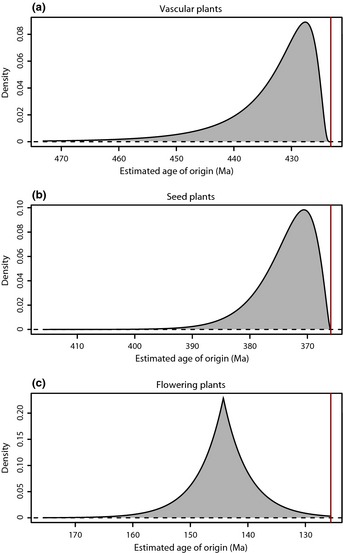
Estimated times of origin (crown ages) for vascular plants, seed plants and angiosperms. The distributions were obtained from modeling the preservation process in PyRate and after combining 100 randomization analyses to incorporate the uncertainties around the ages of the fossil occurrences (see the Materials and Methods section). The vertical red lines indicate the age of the oldest occurrence included in this study for each group. The time of origin of vascular plants (a) was best fitted by a log‐normal distribution with log mean = 2.06, log standard deviation = 0.76, offset = 423.22 million yr ago (Ma; estimated mean age: 433.5). For seed plants (b) the best fit was given by a gamma distribution with shape = 2.44, scale = 3.19, offset: 366 Ma (estimated mean age: 373.8). The time of origin of flowering plants (c) was best modeled by a Laplace distribution with mean = 144.26 Ma, scale 4.36, truncated at 125.65 Ma (estimated mean age: 143.7). These distributions can be directly implemented as calibration priors for dating the phylogenies of these clades using Bayesian relaxed molecular clocks. The parameterization provided here follows that implemented in the popular molecular dating program BEAST (Drummond *et al*., 2012).

The estimated times of origin of vascular plants, seed plants and angiosperms were best fitted by log‐normal, gamma and Laplace distributions, respectively (Fig. 3; Table S3). Our results indicate that the diversification of vascular plants started during pre‐ or Early Silurian times (mean = 433.5; 95% HPD 449.0–424.0 Ma; Fig. 3a). The origins of seed plants and angiosperms were estimated to the Middle Devonian (mean = 373.8, 382.3–367.2 Ma; Fig. 3b) and Late Jurassic–Early Cretaceous (mean = 143.8, 95% HPD: 151.8–133.0 Ma; Fig. 3c), respectively. These ages predate those of the oldest fossil occurrences included in this study for each group, because they reflect the use of all available occurrence data to model the preservation process while incorporating the uncertainties around the fossil ages.

## Discussion

### The dynamics of plant diversification

The balance between origination and extinction is the main factor driving biodiversity dynamics through time (Sepkoski, 1978, 1979; Sepkoski *et al*., 1981), and the only one to be considered in global‐level analyses (migration may play a role at regional scales). This relationship is thus critical to understanding current patterns of plant diversity. Our analyses have shown the main trends of origination and extinction of plant evolutionary history based on global fossil occurrences. From these results, at least two noteworthy observations arise: major mass extinction events had differential impact among plant groups; and strongly negative rates of plant diversification were never sustained through longer time intervals.

There is strong evidence of high extinction rates in terrestrial ecosystems at the Permian–Triassic and Cretaceous–Paleogene boundaries from tetrapods, birds and insects (Benton, 1985; Labandeira & Sepkoski, 1993; Longrich *et al*., 2011, 2012; Fröbisch, 2013). Plant diversification, however, appears to have followed different dynamics. Our results show that the Late Cretaceous biotic crisis, one of the best studied of all mass extinction events, had less effect than expected on plant diversity, despite a supposedly great impact on the configuration of terrestrial habitats due to drastic changes in the world's biota (Krug *et al*., 2009; Schulte *et al*., 2010; Meredith *et al*., 2011). This result supports a recent family‐level analysis of extinction rates for plants, which did not find evidence for significant rate variation at the Cretaceous–Paleogene boundary (Cascales‐Miñana & Cleal, 2014). This boundary led to ecological and functional replacement of life forms, including a total collapse of Paleophytic diversity and a sustained decline of a Mesophytic flora that was progressively replaced by Cenophytic vegetation during the Cretaceous (Cleal & Cascales‐Miñana, 2014).

Interestingly, our results show that the Late Cretaceous is characterized by the lowest net diversification rates in nonflowering seed plants (Fig. 1i), and by negative diversification in spore‐bearing plants (Fig. 1f). In both cases, these patterns are primarily driven by high extinction rates, although in nonflowering seed plants this is further exacerbated by decreased origination rates. The significant increase in extinction rate observed in spore‐bearing plants at the end‐Cretaceous (Fig. 1e) is associated with a collapse of fern diversity from the Paleophytic flora (see Fig. 5 from Cleal & Cascales‐Miñana, 2014). By contrast, positive net diversification rates are reconstructed throughout the Cretaceous for flowering plants (Fig. 1l), which rapidly accumulated generic diversity since the Late Cretaceous (Xing *et al*., 2014). As a result, plant diversity has been dominated by angiosperms since the early Cenozoic, with some contributions from pteropsid ferns and ‘gymnosperms’, mainly pinopsids (Collinson, 1996; Anderson *et al*., 2007; Friis *et al*., 2011).

The sudden increase of extinction rates for nonflowering seed plants in the Late Cretaceous (Fig. 1h) occurs synchronically with a major loss of seed fern diversity. As noted by McLoughlin *et al*. (2008), seed ferns were diverse and abundant during the Triassic–Jurassic time interval, and declined in the Cretaceous synchronically with the radiation of angiosperms. These suggestions are supported by our results (Fig. 1f,l). The almost complete demise of seed ferns (only the Corystospermales briefly survived the Cretaceous–Paleogene event; McLoughlin *et al*., 2008) and the loss of primitive forms of pinopsids (i.e. Cheirolepidiaceae and Voltziaceae) and ginkgoopsids (i.e. Umaltolepidaceae, Leptostrobaceae, Caytoniaceae; Anderson *et al*., 2007), unequivocally caused a major taxonomic shift in floristic diversity. This change, together with the environmental changes following the Chicxulub impact event (Schulte *et al*., 2010), appear to be tightly linked to the success of flowering plants, which immediately after the end‐Cretaceous extinction event underwent a second burst of diversification (Figs 1l, 2d).

The Permian–Triassic mass extinction event is associated with a much more pronounced reduction in plant diversification than observed at the Cretaceous–Paleogene boundary. In fact, the estimated extinction rates following the Permian–Triassic boundary are the highest estimated for all plant groups surveyed (Fig. 1). These results provide further evidence that a substantial depletion of plant diversity took place at the Permian–Triassic boundary (McElwain & Punyasena, 2010), in which, according to Rees (2002), > 50% of all plant species were exterminated. As pointed out by Retallack (2013), the Late Permian mass extinction involved the demise of glossopterids, gigantopterids, tree lycopsids and cordaites. This substantial loss of plant diversity was also recently documented at the family level (Cascales‐Miñana & Cleal, 2014). Moreover, our results show a progressive increase of extinction rates since the middle Carboniferous. This result supports the idea of a gradual floral reorganization, as suggested for South China by Xiong & Wang (2011).

In comparison with the Permian–Triassic and the Cretaceous–Paleogene crises, our results suggest that the other widely recognized mass extinction events (at the Frasnian–Famennian and Triassic–Jurassic boundary) had less detectable effects on plant diversity. However, although Cascales‐Minana & Cleal (2015) found no significant increase in extinction rates at the Frasnian–Famennian boundary at the family level, here we have detected important changes at the generic level. The high extinction rates estimated for the Late Devonian (Fig. 1b) were paired with high origination rates (Fig. 1a), suggesting high rates of taxonomic turnover. Indeed, during this period we observe the disappearance of several spore‐bearing plant groups representing early diverging lineages, such as Zosterophylls (Cascales‐Miñana & Meyer‐Berthaud, 2014). This period is also characterized by the diversification of arborescent lycopsids, early seed ferns and ligniophytes (Wang *et al*., 2005; Galtier & Meyer‐Berthaud, 2006; Meyer‐Berthaud *et al*., 2010; Decombeix *et al*., 2011). The Late Triassic is well documented as the heyday of ‘gymnosperms’ (Anderson *et al*., 2007). However, our results indicate that their highest net diversification may have taken place slightly earlier, in the Middle Triassic (Fig. 1i), followed by significant changes of origination rates across the Triassic–Jurassic boundary (Fig. 2c).

### Origin of major plant clades

The origin of land plants can be established from the Dapingian (middle Ordovician) based on the earliest known cryptospores, whereas the first sporangia is dated from the Katian (late Ordovician) and first robust evidence of macroflora is found at the Wenlock Series (i.e. *Cooksonia*; Wellman, 2014). In comparison, our estimated interval for the origin of vascular plants ranges from 449 Ma (late Ordovician) to 424 Ma (Ludlow, late Silurian) (Fig. 3a). Despite the fact that our inferences are based exclusively on macrofossil evidence, this estimated interval falls within the expected range considering the entire fossil record (i.e. including microfossils) of early land plants. This is consistent with the idea that, especially for early land plants, the spore fossil record is more abundant and less biased than the plant megafossil record because: these early plants produced vast numbers of spores; spores are thought to have primarily accumulated in near‐shore marine environments suitable for sediment preservation; and spores have high fossilization potential due to the fact that they contain sporopollenin, one of the hardest biological materials (Wellman *et al*., 2013). By contrast, the presence of rich assemblages of plant megafossils from early floras is linked mainly to terrestrial environments (Hao & Xue, 2013), which represents a considerable deposition bias. The concordance between our estimates of the origin of vascular plants based on macrofossils and previous estimates based on microfossils highlights the importance of modeling the preservation process when assessing the age of clades (Silvestro *et al*., 2014b). Importantly, due to the delayed appearance of characteristic vascular plant traits in the earliest land plants, the dating presented here suggests that their origin could be even earlier than revealed by the oldest cryptospores.

We placed the origin of seed plants between the Givetian (late Middle Devonian), and the Famennian (Late Devonian). Our estimation slightly predates that derived by Anderson *et al*. (2007), which could be expected considering the inherent constraints of identifying fossil‐genera. A critical element here is the controversial assignment of fern‐like foliage into spermatophytes. For instance, the genus *Pecopteris* has mainly been used for ferns. Only one seed fern has been placed in that genus, but is now referred to as *Dicksonites* (Callistophytaceae; Galtier & Bethoux, 2002). Indeed, it was recently documented that the type of *Pecopteris* is a fern of the extinct family Tedelaceae, and the generic name should be restricted to that family (Cleal, 2015). Similarly, *Sphenopteris* has been regularly used for both ferns and seed ferns. However, the type is a Mississippian age seed fern belonging to the Lyginopteridales (C. J. Cleal, pers. comm., 2 May 2014).

Darwin described the appearance of early angiosperms as an abominable mystery due to their ‘recent’ appearance in the Cretaceous fossil record, their apparent speed to adopt ‘modern looking’ leaves, and their current ecological dominance (Friis *et al*., 2010). Although our knowledge about angiosperm radiation has significantly advanced since the 19^th^ Century, there is still considerable controversy about the timing of angiosperm origin. The root of this debate is derived from different scenarios obtained from using macro vs microfossil evidence or paleontological vs molecular data. From the fossil record, Friis *et al*. (2011) argue that there is no unequivocal evidence of angiosperms before the Cretaceous. Nevertheless, other authors have suggested an early Jurassic (201.3–174.1 Ma) origin based on macrofossil evidence (Wang *et al*., 2005, 2010). An even earlier origin of angiosperms has been proposed based on pollen data, dating back to the Anisian (Middle Triassic, 247.2–237 Ma) (Hochuli & Feist‐Burkhardt, 2013). In summary, pre‐Cretaceous fossil evidence of angiosperms has been considered either equivocal or plagued with problems of geological dating, with no consensus reached to date.

We find further discrepancies in the timing of angiosperm origins based on molecular evidence through phylogenetic dating analyses. As summarized by Doyle & Endress (2010), Bell *et al*. (2010) estimated that angiosperms originated *c*. 183–147 Ma (early to late Jurassic), whereas Smith *et al*. (2010) proposed older estimates ranging between 228 and 217 Ma (Late Triassic). A very wide interval is provided by Magallón *et al*. (2013) who, based on different analyses, estimated that angiosperms originated between 258 Ma (Late Permian) and 158 Ma (Late Jurassic; see also Magallón, 2010; Magallón *et al*., 2015). Our results suggest an angiosperm origin between 152 and 133 Ma, centered at 143.8 Ma (Fig. 3c), thus assigning the highest probabilities towards the boundary between the Late Jurassic and the Early Cretaceous. Our estimation derives from an analysis of the entire set of macrofossil occurrences and accounts for the preservation process.

Although the direct incorporation of fossil taxa in molecular dating is possible and promising (Ronquist *et al*., 2012; Heath *et al*., 2014), these methods either require large morphological datasets or are computationally intensive and potentially difficult to implement on large clades. Thus, many studies still rely on fossil prior calibrations for molecular dating. The methodology used in this study constitutes a novel approach to objectively define calibration priors, in a process‐based and data‐driven analysis that is able to handle the inevitably incomplete nature of the fossil record. The age estimates derived here for the three major plant clades (Fig. 3) could thus be directly used as prior crown age distributions of those clades in molecular dating analyses.

### Conclusions

Our results have provided new insights into the origin and diversification of vascular plants. This represents the first Bayesian analysis of their diversification dynamics, based on publicly available plant macrofossils at a global level, and separating the three major groups of plants recognized today. We found that plants underwent important phases of extinction across three of the ‘Big Five’ extinction events (at the Frasnian–Famennian, Permian–Triassic and Cretaceous–Paleogene boundaries), which represented key points of floral turnover and contributed to major diversity changes of plant life. However, there was a differential effect of those events on each plant group analyzed, and substantial differences in the impact of each event on diversification dynamics. Although many uncertainties remain regarding the age of origin of major plant clades, our analyses have provided new estimates that help in reconciling current fossil evidence with a robust probabilistic approach that takes into account a complex and variable preservation process.

## Supporting information

Please note: Wiley Blackwell are not responsible for the content or functionality of any supporting information supplied by the authors. Any queries (other than missing material) should be directed to the *New Phytologist* Central Office.

**Table S1** Raw data downloaded from Paleobiology Database

**Table S2** Complete reference list of the original download

**Table S3** Fit of different distributions to the posterior estimates of the origination times of three plant groups calculated by Akaike Information Criterion
